# Understanding “interests”: historical insights for managing conflicts of interest in healthcare and biomedical science

**DOI:** 10.1007/s11019-025-10268-5

**Published:** 2025-04-22

**Authors:** Miriam Wiersma, Ian Kerridge, Wendy Lipworth

**Affiliations:** 1 Faculty of Medicine and Health, Sydney School of Public Health, Sydney Health Ethics, The University of Sydney, Rm 134, Edward Ford Building A27, Sydney, NSW 2006 Australia; 2 Haematology Department, Royal North Shore Hospital, Reserve Road, St Leonards, Sydney, NSW 2065 Australia; 3 Ethics and Agency Research Centre, Macquarie University, 75 Talavera Rd, Macquarie Park, NSW 2109 Australia

**Keywords:** Interest, Conflict of interest, Medicine, Pharmaceutical industry

## Abstract

Conflicts of interest are widely regarded as being morally, socially, and scientifically problematic in the many sectors, including in the health sector. There has been considerable attention paid to managing conflicts of interest in clinical practice, medical research and health policy through strategies such as recusal, disinvestment, and disclosure. While these efforts have been important, they are often based on a superficial account of “interests”, as few in healthcare and biomedical science have sought to unpack the concept. In this paper, we argue that adopting an historically and philosophically informed account of interests can enrich our thinking about COI in healthcare and biomedical science, and lead to the improvement of COI management strategies. To support this claim, we first provide an overview of contemporary debates about COI in these domains. We then summarise the historical trajectory of the concept of “interest” and show how these insights can be used to inform the management of COI in healthcare and biomedical science using the example of physicians’ relationships with the pharmaceutical industry. In particular, we challenge assumed hierarchies of interests and call for increased attention to the multiplicities of interests, both financial and non-financial, that may at times converge and conflict.

## Introduction

Conflicts of interest (COI) have long been a focus of philosophical and professional concern, including in healthcare and biomedical science. In these sectors, there have been extensive and sustained efforts to address the problem of COI, but there is ongoing disagreement about how best to define, identify, and manage conflicts of interests. Questions include whether there is a clear hierarchy of interests and whether there needs to be a hierarchy of interests for a COI to exist; whether non-financial COIs should receive as much attention as do financial COIs; and how normative we ought to be about interests and COI. We explore each of these debates briefly below.

### Is there a clear hierarchy of interests in medicine and biomedical science?

There is broad agreement about the importance in medicine of giving high priority to the interests of patients. This view is reflected in declarations, statements and oaths, such as the Declaration of Geneva’s statement that “the health and well-being of my patient will be my first consideration” (World Medical Association 2020). The commitment to patients’ interests also forms an integral part of the social contract between physicians and society—whereby medical professionals receive status, respect, financial reward, autonomy in practice and the privilege of self-regulation in exchange for prioritising patient and societal interests above their own (Cruess and Cruess 2020, 2008; Doernberg and Truog 2023; Brody and Doukas 2014). By framing these moral and altruistic expectations as professional ethical obligations, the social contract serves as the basis for medical professionalism and therefore, both guides physician behaviour and establishes the profession’s responsibilities to patients and society (Pellegrino and Relman 1999; Swick 2000; Brody and Doukas 2014).

The primacy of patients’ interests is sometimes justified on the basis of a “fiduciary relationship” that acknowledges the innate power differential between physicians and patients and the role of physician as the patient’s agent (Ludewigs et al. 2022). Some reject the notion of a fiduciary relationship between physicians and patients because it does not capture the precise legal nuances of the physician–patient relationship in some jurisdictions (Mayes 2020). However, there is broad agreement that physicians are obligated to use their specialised knowledge and skills in service of their vulnerable patients' interests (Ludewigs et al. 2022).

While there is little disagreement about whether patients’ interests should be given high priority, there is disagreement as to whether this translates into a *hierarchy of interests* that can be defined independently of context.

Many people believe that such a hierarchy exists. For example, the most widely used definition of conflict of interest (COI) in medicine is that presented in a 2009 Institute of Medicine document entitled “Conflict of Interest in Medical Research, Education and Practice” (Lo and Field 2009) which defines COI as “a set of conditions in which a professional judgement concerning a *primary interest* (e.g. a patient’s welfare or scientific integrity) tends to be unduly influenced by a *secondary interest* (e.g. financial gain)” (emphasis added). According to this definition, which is derived from the work of Dennis Thompson (Thompson 1993), primary interests are those “determined by the professional duties of a physician or scholar”, whereas secondary interests include relationships, financial incentives or the desire for prestige or power (Thompson 1993). Thompson takes care to emphasise that secondary interests are not illegitimate and may, in fact, be an important part of professional practice—however, they become problematic when they unduly compromise or appear to compromise primary interests (Thompson 1993). Importantly, this definition assumes a hierarchy of interests that can be known and agreed upon, in abstract terms—for example, a physician’s primary interest is expected to be patient care, with all other interests placed secondary to this (Mayes 2020; Stead 2017; Geiderman et al. 2017; Thompson 1993).

Notwithstanding the popularity of this definition of COI, we and other scholars have questioned whether there can be a pre-defined hierarchy of interests in healthcare and biomedical science (Komesaroff et al. 2019). There are two reasons for this: first, while physicians have broadly recognisable social contracts with society, their roles are complex, involving not only patient care, but also administration, bureaucracy, teaching, research, compliance, and so on. Modern physicians are accountable not only to patients and society, but also to governmental and commercial institutions which oversee their performance, competency, productivity, and the cost-effectiveness of their treatment decisions (Cruess and Cruess 2008). They also have obligations to colleagues, students and research participants.

For example, a physician engaged in research needs to attend to the fundamental goal of medical research, which is to produce generalisable knowledge and advance individual and public health, rather than to seek therapeutic benefit for individual patients (Miller and Brody 2003; Doernberg and Truog 2023). While a physician-researcher cannot knowingly place a patient at undue risk, they can and do subject them to some risk, as well as to inconvenience and pain. Responsible physicians manage these dual interests by taking steps to ensure informed consent, minimise risk, maintain scientific integrity, and protect vulnerable research participants while pursuing knowledge that will advance medical science (Brody and Miller 2003).

There is an extensive literature on such “conflicts of commitment” and “dual agency dilemmas” and ongoing debates about how they should be managed (Tilbert 2014; Doernberg and Truog 2023). The fact that these debates continue underscores the complexity of physicians’ roles and the associated social contract. Furthermore, the nuances of physicians’ commitments to patients and society are constantly reshaped in response to societal and cultural shifts (Cruess and Cruess 2008, 2020; Wynia 2008; Brody and Doukas 2014). For example, the Covid-19 pandemic required shifts in the delivery of healthcare, for example, replacing face-to-face consultations with telehealth consultations that were not necessarily in individual patients’ best interests but (arguably) protected the community from harm.

A second reason to question the existence of a pre-defined hierarchy of interests is that even a physician’s *own personal* interests can sometimes override their obligations to patients. For example, there is an extensive literature on “conscientious objection in healthcare” that explores the question of whether it is permissible for physicians to privilege their own beliefs over the needs and desires of patients (Giubilini and Savulescu 2020). Additionally, there is a body of literature on the “duty to care” that considers when it is acceptable for physicians to refuse to care for patients when doing so might harm them—for example, by exposing them to an infectious disease (Lipworth 2020).

A more prosaic, but undoubtedly common scenario might be a physician who is involved in non-life saving patient care (e.g. writing scripts/admitting/clerking a patient/giving education to a patient) is contacted by a very distressed child (their own) who needs their immediate attention. In this situation even though the physician is involved in direct patient care, and even though there is a loose social contract that privileges patient care, it is not the case that this does or should take a priori priority over other non-financial interests. Rather, the physician will weigh them up, put in place mitigation strategies if needed to avoid harm, and then privilege their own child.

Even financial interests may sometimes be prioritised over patients’ best interests. As long as we accept any kind of privatised healthcare, the reality is that money is being put before patients, who would always be better off if clinicians offered their services for free. As uncomfortable as it may be, it cannot be said that there is a simple hierarchy in which financial interests are always situated beneath patient care. Of course, any sort of price gouging, or failing to provide the best possible care simply because this generates more profit, is morally objectionable, but this does not mean that there is a simple hierarchy of interests in which financial interests are always and completely subordinated. While it is never justifiable for a physician or researcher to knowingly harm or disadvantage a patient or research participant for financial gain it does not follow from this that a simple a priori hierarchy can be established in which patient care *always* and *in every way* comes before financial gain.

Physicians also increasingly work for large corporations (Cruess and Cruess 2008, 2020; Wynia 2008). This shifting dynamic, driven by profound changes in healthcare's structure and financing, has prompted researchers to advocate for a renegotiated social contract that acknowledges medicine's increasing commercialisation and commodification (Wynia 2008; Cruess and Cruess 2008, 2020). Although there is broad consensus that healthcare's primary purpose should remain focused on patient care rather than profit generation (Brody and Doukas 2014), researchers have claimed that we need to explicitly acknowledge and address the fact that physicians navigate competing pressures between financial considerations and optimal patient care (Wynia 2008; Wendler 2010). This recognition forces us to reexamine the traditional principle that patient interests should always come first, as physicians increasingly need to balance these interests against other systemic demands and constraints (Wendler 2010).

This perspective on social contracts, physicians’ roles and the realities of health care delivery challenges the utility of definitions of COI based on presumed hierarchies of interests—including the widely used definition proposed by Thompson (Thompson 1993).

### Can conflicts of interest exist if there is no hierarchy of interests?

A related debate is whether there needs to be a clear hierarchy of interests in order for a conflict of interest to logically exist.

Australian philosopher and ethicist Christopher Mayes describes the increasing commercialisation of healthcare and the growing lack of clarity about physicians’ obligations. To illustrate this point, he offers the example of an Australian In Vitro Fertilisation (IVF) company that states in its Code of Conduct that physicians are required to act in “the best interest of the Company” and that “your primary responsibility is to the Company and its Shareholders as a whole” (Mayes 2020). Mayes does not condone this state of affairs; rather his point is that a desirable state of affairs in which patients' interests are always given primacy is giving way to a less desirable one in which commercial interests are given increasing weight. Mayes not only laments the loss of moral clarity, but also maintains that if this trend is to continue, the very meaning of ‘conflict of interest’ will be lost. He argues that if it becomes unclear that there is a clearly known and agreed upon primary interest for physicians due to the impact of market logics on medical professionalism and practice, the concept of a conflict of interest can no longer be applied (Mayes 2020).

An alternative view is that there is a logical error in this reasoning. While it may be easier to identify conflicts of interests when a hierarchy of interests exists, conflicts can still exist between interests that are not hierarchically organised. For example, it may be that there is no clear hierarchy between a physicians’ interest in conserving scarce healthcare resources and offering the best possible healthcare. But this does not mean that there is no “conflict of interest” in such circumstances if we understand interests as personal concerns or obligations linked to social roles, and conflicts of interest simply as circumstances in which two concerns or obligations conflict (Komesaroff et al. 2019).

### Should more attention be paid to non-financial conflicts of interest?

It is widely recognised that interests and conflicts of interest can be both financial and non-financial. Financial interests are those interests relating to monetary gain, including payment for services; speakers’ fees; paid board membership; paid legal representation; and ownership of, or equity in, companies (Hakoum et al. 2016; Shawwa et al. 2016; DeCensi et al. 2018). Non-financial interests are both diverse and extensive—including personal relationships; religious, political or personal beliefs; desires (such as the desire for status, respect and reputational enhancement) as well as role-related obligations or duties (such as a physician’s obligation to act in the best interest of their patient) (Bosch et al. 2013; Wiersma et al. 2018a, b).

Although less empirical research has been conducted into the impacts of non-financial COI than into financial COI, growing evidence indicates that non-financial interests and COI can be problematic for physicians and researchers (Viswanathan et al. 2014; Abdoul et al. 2012; Wiersma et al. 2020; 2024). Our recent scoping review of non-financial COI in health-related journals found that many believe non-financial COI are problematic—drawing attention to belief or viewpoint related COI (e.g. political or religious beliefs), career-related COI (e.g. career advancement), interpersonal COI (e.g. the desire to get along with or impress others) and status related COI (e.g. the desire for prestige or fame) (Wiersma et al. 2024).

While few deny the existence of non-financial interests and conflicts of interest, there is ongoing debate in healthcare and biomedical science about whether or not non-financial COI require attention and management alongside financial COI (Bero 2014; Lenzer 2016; Rodwin 2018; Wiersma et al. 2018a, b). Although journals and funding bodies increasingly ask for declarations of non-financial interests and conflicts of interest, there is some push-back, with critics arguing that financial conflicts of interest are more objective, quantifiable, ethically problematic and manageable than their non-financial counterparts (Bero 2014; Garattini and Padula 2019; Garattini et al. 2020; Lenzer 2016). Marc Rodwin, for example, describes how in contemporary law the only COI that are regulated are those relating to financial interests (Rodwin 2017). These financial COI, he claims, can be “easily” avoided or eliminated—unlike other interests, such as intellectual interests (e.g., intellectual commitments, the desire for career advancement or reputational benefits) (Rodwin 2018, 2017). He criticises recent attempts to redefine COI in medicine to included non-financial interests because they are confusing and lack practical utility (Rodwin 2018, 2017).

Others have argued that labelling personal attributes or beliefs, relationships and intellectual commitments as (non-financial) COI “muddies” the very concept of COI, and makes COI appear so ubiquitous that they cannot be meaningfully managed (Bero et al. 2016; Grundy et al. 2020). These researchers also claim that focusing on non-financial COI has several negative consequences: it diverts attention from problematic financial COI, obscures issues around diversity and representation, and risks causing discrimination within biomedicine (Bero et al. 2016; Grundy et al. 2020).

We and others have countered this position, arguing that non-financial COI need attention because they are highly influential drivers of behaviour that have the potential to come into conflict with other interests (Goldrick 1994; Marshall 1992; Pellegrino 1992; Horrobin 1999; Levinsky 2002; Wiersma et al. 2018a, b; Bion 2009; Detsky 2006; Shaw 2014; Malay 2016; McKinney and Pierce 2017). Indeed, some have noted that non-financial interests such as reputational enhancement, status, prestige and public recognition, may in fact, be more powerful drivers than financial interests (Kirkpatrick et al. 2012). Furthermore, financial and non-financial interests and COI are often intertwined, and it is possible that strategies to manage financial COI can be carefully adapted to address the complexity and diversity of non-financial COI (Wiersma et al. 2018a, b).

### Should COIs be presumed to be morally wrong?

A fourth debate about COI in healthcare and biomedical science concerns how normative (i.e. negative) we ought to be about conflicts of interest in medicine. There is common perception within medicine that COI are inherently ethically problematic (Horton 1997; Rosenberg 2017; Johnson 2004). This differs from the ways in which COI are judged in some other domains, such as in legal practice, where conflicts of interest are seen to be rule based, structural and objective (Johnson 2004). For a lawyer, there is no shame in having a COI—it is simply something to be managed (Johnson 2004). For a physician, however, an accusation of COI may lead them to believe that they have made an ethically questionable decision, which may reflect poorly on their character (Johnson 2004). There are several reasons why there are negative associations with the term COI in medicine. These include media attention to high profile cases involving physicians, researchers and policymakers with COI, (Owens 2013) and growing evidence of the impact of financial incentives on their behaviour (Rosenberg 2017; Brax et al. 2017).

Some scholars and commentators support this negative perception of COI in healthcare and biomedical science, both because of the dangers posed by COI and because of what they perceive to be the potentially corrective effect of social stigma (Angell 2009). Others, in contrast, have argued that it is unfair to negatively judge people who have COI (Rosenbaum 2015). This is because the complexity of contemporary social and professional roles means that the existence of conflicts of interest “must now be recognised—for better or worse—as one of the distinctive features of modern life”, (Komesaroff et al. 2019, p. 577) and because people may be deterred from disclosing COI if the assumption is that having interests and COI is *ipso facto* unethical (Horton 1997; Rosenberg 2017; Maj 2008). This has led some to argue that, rather than using stigma to deter professionals from having COI, we should “normalise” COI and accept and encourage open discussion about the multiple directions in which professionals can be pulled (Horton 1997; Leas 2016; Clark 2015).

## A philosophical account of ‘interests’

Contemporary accounts of COI in medicine, and debates about their definitions, normative status and scope, tend to situate themselves in recent history—often identifying the aforementioned Institute of Medicine report as the starting point of thought in this area. With a few exceptions, (Komesaroff et al. 2019; Abraham 1995; Mayes 2020) there is little or no reference to the long history of discussion of interests in the philosophical, sociological and economic literature.

We believe that this is a significant lacuna, and that a rich, philosophically informed account of interests can enrich our thinking and address areas of contestation regarding COI in healthcare and biomedical science. To address this gap, in the following sections we trace the historical trajectory of the concept of interest, drawing on historical work by Albert O Hirschman, Richard Swedberg and J. A. Gunn and apply it to contemporary discussions (Hirschman 1987; Hirschman 2013a b; Swedberg 2005a b; Gunn 1968;).

In modern everyday language the term “interest” is used to describe distinct phenomena across diverse contexts. In economics, for example, the term interest is used to describe the fee paid to borrow a sum of money or the amount of ownership a stockholder has in a company (Oxford English Dictionary 2024). In legal contexts, the term is used to describe an individual or group having a legal concern—particularly a title or right to property (Oxford English Dictionary 2024). More generally, however, the term interest (as a noun) is used to describe an object, place, person, subject or phenomenon “in which an individual has an interest or concern” (Oxford English Dictionary 2024). These types of interests may relate to both concrete entities (such as land) and more abstract phenomena (i.e. that which has no physical basis or form—e.g. freedom or justice) and it is the latter that form the focus of this historical review.

The word “interest” originally came from the Latin phrase “inter esse” (Heilbron 2015), meaning to “be between” and “to make a difference” (Swedberg 2005b). When the term first emerged in the fifteenth century throughout Europe, it was used as a euphemism for usury (Hirschman 2013a; Heilbron 2015). To have an interest within this early use of the term, was to have some form of right, title or claim to property, (Eastwood 2005) demonstrating the early legal and economic origins of the term. It is noteworthy that, given the association with usury, the term interest during this period had negative connotations (Hirschman 2013a; Heilbron 2015).

In the sixteenth century, the term interest took on a more generalised meaning, referring to drivers of human behaviour, including those of the Ruler of the State (i.e. “princely interest) (Swedberg 2005a; Heilbron 2015; Hirschman 2013b). Interests were perceived to be diametrically opposed to the passions—that is, interests were seen to be rational and calculating, whereas passions were seen to be wild and destructive (Swedberg 2005a; Heilbron 2015; Hirschman 2013b). Interests could be used, therefore, to tame the passions, and rulers were expected to govern by interest and not be influenced by passions, desires or others’ opinions, as the Duke of Rohan (1579–1638), wrote:

“In matters of State, one ought not to…be led by ordinate desire, which carry us oftentimes to take things beyond our strength, nor by violent passions which do…trouble us, nor by superficial opinions, whereby ill-conceived scruples are ministered onto us, *but rather by our proper Interest, guided by reason alone*”. (Rohan 1663)

During the late 16th and early seventeenth century, the concept of interest came to be regarded as having both positive and negative connotations. Interests continued to be viewed as a force through which to temper the destructive passions (Hirschman 1987), but self-interest (referred to by some as “private interest”) came to be associated with greed—which underpinned a somewhat pessimistic view of humankind as being driven solely by self-interest (Hirschman 1987).

The concept of interest flourished in the seventeenth century, which was to become the “peak of the interest doctrine” (Hirschman 1987). During this period, the passions continued to be viewed as dangerous and destructive—a force that could only be controlled by the pursuit of rational interests. During this time, the political maxim, “interest will not lie” gained popularity throughout England (Gunn 1968; Swedberg 2005b). The maxim was normative, in that it prescribed how a Ruler ought to behave, i.e. through the awareness of their interests and the logical pursuit of these, and that this would result in political stability and social order (Gunn 1968; Swedberg 2005b).

At the same time, there was broad recognition that all members of society have interests, and the concept moved from the political realm into that of the general public (Gunn 1968). During this period, several intellectuals, including French Huguenot, the Duke of Rohan and English journalist, Marchamont Nedham, explored the notion of interests as drivers of human behaviour and questioned their supposed moderating impact on the passions. The French moralist Francois de la Rochefoucauld (1613–80) captured the subtlety of interests (Swedberg 2005b; Rochefoucould 1665):

“Interest speaks all sort of tongues and plays all sorts of characters; even that of disinterestedness”.

de La Rochefoucauld argued that interests underpin all human behaviour, even when people deny that this is the case (Swedberg 2005b; Rochefoucould 1665). He was also careful to note that interests are neither a force for good nor bad—rather, “interest sets at work all sorts of virtues and vices”, and that “interest blinds some and makes some see” (Rochefoucould 1665). Interests, therefore, could drive an individual towards action, or alternatively, could stand in the way of an individual attaining their goals (Swedberg 2005b).

During the 18th to early nineteenth century, the concept of interest underwent considerable transformation. Whereas previously “rational” interests were seen as normatively good and self-interest as problematic, in the early eighteenth century, individuals’ pursuit of their own interests was legitimised and viewed as having positive societal and economic benefits. The notion of “doux commerce” also rose in popularity during this time, in which commerce was viewed as having a powerful civilising effect on society. Adam Smith (1723–1790) in his famous notion of the invisible hand, removed any lingering negative associations with interest and interest driven behaviour (Hirschman 2013a; Smith 1776, 1759). The invisible hand described how individuals’ self-interested^1^ behaviour can lead to unintentional benefits for society:

“He attends his own gain, and he is in this, as in many cases, led by an invisible hand to promote an end which was no part of his intention” (Smith 1759, p. 184).

Smith maintained that individuals provide services not out of concern for others, but out of their own self-interest—in particular, their interest in economic gain. This served to legitimise the pursuit of economic self-interest and to remove the guilt once associated with the pursuit of pecuniary gain, as acting in one’s interest would lead to benefits for all of society (Hirschman 2013a). Consequently, interest (even in the self-interested sense) took on a more positive meaning as it was no longer closely associated with selfishness and greed (Hirschman 2013b).

For Smith, interests were not the sole driver of human behaviour, rather self-interest was one of several important drivers along with passions (e.g. ambition), drives (e.g. hunger) and emotions (Barbalet 2012; Black 2006). Smith claimed that it was through appeals to individuals’ self-interest that economic cooperation occurs, which in turn, serves to benefit society (Barbalet 2012; Black 2006). For Smith, self-interest was not unproblematic—if unrestrained, self-interest driven behaviour could negatively impact upon others. Sympathy, Smith argued, was therefore required to moderate self-interest, and help individuals to be compassionate towards others (Smith 1759).

From the early nineteenth century, the notion of interests in relation to human behaviour and decision-making was a key concern for utilitarianism,^2^ appearing in the works of both Jeremy Bentham (1748–1832) John Stuart Mill (1806–1873) (Heilbron 2015). The exploration of interests as potential drivers of human behaviour was an important aspect of both theorists’ work. Bentham claimed that individuals were primarily driven by self-interest, namely, the pursuit of pleasure and avoidance of pain (Heilbron 2015; Swedberg 2005b). He also developed the idea of “sinister interests”, later adopted by Mill, which were interests that were incompatible with the public good (Gunn 1986).

In “*The Table of the Springs of Human Action*”, Bentham developed a comprehensive categorisation system of different pleasures and pains, as well as 14 corresponding interests and motives (Bentham 1817). Bentham proposed that pleasure, pain and interests acted as “springs to actions” via neutral, positive, or negative motives. For example, the so-called “interest of the spying glass” (associated with curiosity) had the corresponding neutral motives of curiosity or love of novelty, and negative motives of meddlesomeness or disrespect. Pecuniary interests or “the interest of the purse” were associated with neutral motives—the desire for wealth; negative motives—avarice, greed and corruption; and positive motives—frugality and thriftiness (Bentham 1817). Although criticised as being a tautological approach to interests, (Swedberg 2005b) the *Table of the Springs of Action* offers a thought-provoking approach to understanding interests, motives, and human behaviour. Bentham’s theory reminds us of the diversity of interests and that the pursuit of a specific interest may result in a neutral, positive or negative outcome.^3^

Like Bentham, Mill believed that individuals were motivated by self-interest, in particular, economic self-interest (Hirschman 2013a; Swedberg 2005b). This was most clearly seen in the notion of “homo economicus” (i.e. economic man), which was widely attributed to Mill, although he never used the term (Ng 2008; Swedberg 2005b; Persky 1995). “Homo economicus” is the idea that humans are driven solely by economic self-interest and preferences and have the rational capacity to act in such a way so as to maximise these preferences (Mele 2014). While Mill acknowledged the role of other interests, such as leisure and procreation, they were secondary to economic interest (Hinnant 1998).

Due to his focus on economic self-interest, Mill came under criticism for developing a theory of human behaviour that was overly narrow (Hinnant 1998). Such criticism, however, failed to recognise that Mill himself argued that “political economists don’t deny other motives, but merely abstract from them” (Mill 1848) and that while the idea of homo economicus was a radical simplification, it was necessary for the emerging discipline of economics (Hirschman 2013a; Smelser and Swedberg 2005a, b). His focus on economic self-interest was one of several factors during the nineteenth century that contributed to the narrowing of the concept of interest to economic self-interest.

In the late 19th to early twentieth century, the concept of interest drew the attention of sociologists and influential scholars including Karl Marx, Max Weber and Albion Small (Swedberg 2005a, 2005b; Heilbron 2015). During this time, sociologists delved deeply into the relationship between individual interests and social life to develop a sociological concept of interest (Swedberg 2005a). Several opposing attitudes towards the role that interests had in driving human behaviour emerged in sociological discourse—the first that perceived interests as the sole driving force of human action, (Small 2019; Swedberg 2005a) and the second view which held that there were multiple drivers of behaviour, including interests (Swedberg 2005a).^4^

In contrast to Adam Smith, who claimed that individual pursuit of self-interest within a free market would be of broad societal benefit, Karl Marx (1818–1883) argued that the unrestrained pursuit of self-interest would lead to class conflict and social disruption (Ritzer 2003). While other theorists focused on the interests of individuals, Marx concentrated on the collective interests of social classes and introduced the concept of “class interest” (Swedberg 2005a; Berlin 2013). For Marx, material (i.e. economic) interests drove human behaviour, and these interests were attached to economically determined and historically constituted social positions (Lizardo and Stoltz 2017; Berlin 2013; Barnes 2015).

In order to understand human behaviour, therefore, Marx argued that one must attend to the broader social and economic context—including the interests of different social classes, the social class structure as a whole, and ways in which different classes come into conflict with one another (Berlin 2013). For Marx, it was purely economic interests that mattered, as different social classes would act according to what is in their economic interest—whether it was in their economic interest to maintain the status quo, or whether it was in their interest to rise up against the existing social structure (Berlin 2013; Spillman and Strand 2013). Importantly, therefore, human behaviour driven by economic interests and ideas had the power to enact profound social change (Barnes 2015).

Max Weber (1864–1920) also claimed that interests were a significant driver of human behaviour (Swedberg 2005a). According to Weber, there were two broad types of interests, material (or base) interests—including survival, food, and sex, and ideal interests—including intellectual pursuits, religious motivations and relationship-based interests (Swedberg 2005a; Swedberg and Agevall 2016; Lizardo and Stoltz 2017). Material interests were constant over time and consequently shared by all individuals, whereas ideal interests were subject to historical variation and dependent upon the broader social context (Lizardo and Stoltz 2017). Weber claimed that material and ideal interests were equally potent, (Swedberg and Agevall 2016) and that ideal interests, could at times, be even more powerful drivers of behaviour than economic interests (Swedberg 2005a). Weber rejected the notion that there was a single driver of behaviour and proposed a multicausal model of human behaviour as being shaped not only by interests, but also ideas, values, emotions and social conventions (Swedberg and Agevall 2016; Swedberg 2005a).

Other sociologists also traced all human behaviour back to interests, including, American sociologist Albion Small, (1854–1926) and his predecessor Austrian sociologist Gustav Ratzenhofer (1842–1904) (Swedberg 2005b). According to Small, interests were “affinities, latent in a person, pressing for satisfaction” which individuals may or may not be aware of (Small 2019). He suggested that there were six general classes of interests: health, wealth, sociability, knowledge, beauty and rightness. All human behaviour, according to Small, was driven by these interests, or some combination thereof (Swedberg 2005b).

The argument that all human behaviour was driven by interests was not unproblematic, and proponents of this view faced growing criticism that their claims were tautological (Hirschman 1987, 2013b) and diluted the concept of interest as it was used to explain all human behaviour (Hirschman 1987, 2013b). This was one of the reasons why there was a move away from the concept of interest during the nineteenth century, along with a collapse of the boundary between passions and interests; a questioning of the innocence of economic self-interest; as well as the emergence of the new science of economics (Hirschman 1987, 2013b).

Whereas in the seventeenth century the passions were viewed as a destructive and powerful force that needed to be controlled by rational and calculated interests, the Romantic era (late eighteenth century–nineteenth century) served to recast passions in a more positive light as potent sources of creativity (Hirschman 2013b). As attention was drawn to more emotive sentiments such as sympathy and generosity, interests, as Hirschman explains, “no longer looked so attractive” (Hirschman 2013a, b p 204). Another reason why the concept of interest fell from popularity was the questioning of the notion of “doux commerce”, and growing recognition that the pursuit of economic self-interest could be highly problematic (Hirschman 2013a). While the drive for pecuniary gain had once been viewed as innocent and having numerous benefits for society, during this period it was recognised as a powerful and potentially destabilising force (Hirschman 2013a).

The emergence of the new science of economics in the nineteenth century, saw a move away from the rich and complex sociological analyses of interests—with interest ultimately reduced to a narrow economic concept (Barbalet 2012; Swedberg 2003, 2005b). While other emerging social sciences, including psychology and sociology, began to adopt the perspective that human action was largely instinctive and nonrational, such an assessment was simply incompatible with the economic view of human behaviour being driven by rational economic self-interest (Hirschman 2013a). Economics, therefore, retained its narrow conceptualisation of interest, which was later adopted by other social sciences and remains widely in use today (Hirschman 1987; Swedberg 2005b; Heilbron 2015).

This limited economic definition of interests, however, has been rejected by some contemporary philosophers, including influential American legal philosopher Joel Feinberg (1926–2004) (Feinberg 1987). Feinberg outlines a sophisticated account of interests in his analysis of legal harm and presents a framework of human interests to identify situations where the law can legitimately restrict an individual’s liberty (Feinberg 1987). While an analysis of Feinberg’s rearticulation of the harm principle and the role interests play in guiding legal action is beyond the scope of this paper, it is useful to briefly explore his definition and framework of interests.

Feinberg defines interests as consisting of “all of those things in which one has a stake”. (Feinberg 1987, p 34). Feinberg argues that there are two kinds of interests: “welfare interests” are those interests that are necessary for an individual to exist and experience a very basic level of wellbeing or functioning. They include an interest in one’s physical, psychological and social functioning, as well as financial and physical security. “Ulterior interests” are an individual’s higher order aspirations and goals, for example, intellectual or creative pursuits, relationship goals or the pursuit of hobbies. Within this conceptualisation, interests can be both financial (such as the desire for financial security) or non-financial (such as the desire for health) (Feinberg 1987).

In his “interest network”, Feinberg describes the complex relationship between passing and instrumental wants, welfare interests and focal aims. Passing wants are temporary desires (e.g. the desire for an ice cream) that will not result in harm if they are not met, as the individual’s interests will not be affected. Instrumental wants are the desires an individual has (e.g. working late) that are simply the means to a more permanent interest or goal (e.g. financial security). Focal aims are the core life goals created by an individual’s ulterior interests—they are stable and deeply desired wants that an individual can influence through their actions (Feinberg 1987).

Welfare interests provide the foundation needed to pursue ulterior interests and focal aims, while passing and instrumental wants may either support or potentially conflict with both welfare interests and focal aims. At the individual level, part of practical reasoning involves managing the potential conflict between wants, interests and goals. Where one individual’s interests, particularly their welfare interests, are wrongfully set back by another individual, group or individual, this constitutes legal harm and legal redress is warranted (Feinberg 1987). Feinberg's work on interests and the harm principle has been influential in contemporary debates about the relationship between interests, rights and harm, as well as the limitations of liberty and the role of the law (Lieberman 2012).

## Application to contemporary debates about conflicts of interest

This historical survey of interests shows that there have been many different accounts of the concept, including regarding their normative status and the corresponding status of their counterpoint—the passions; the degree to which they explain human behaviour; their breadth (i.e. whether or not they are purely economic); whether they are stable and universal or malleable and contextual; and whether they reside in individuals or collectives.

Although those debating the management of interests (and conflicts of interest) in healthcare appear largely unaware of this historical trajectory, many of the current debates about COI in healthcare have residues of historical disagreement and may be advanced by recognising this. While historical accounts of how terms have been used are not arguments in and of themselves for ongoing thinking or practice, history can help to buttress other arguments by showing the utility, over time, of particular ways of conceptualising and applying understandings of interests.

### Hierarchies of interests

The debate about whether there needs to be a hierarchy of interests in order for a conflict of interest to exist rests in part on whether one believes that interests themselves exist in a hierarchy of importance or value. As early as the eighteenth century there were discussions about whether certain interests ought to be prioritised over others (Helvetius 1758; Wootton 2001). Claude Helvetius, for example, described how in the context of multiple and potentially divergent interests, the Ruler of State, should take care to ensure that their interests were aligned with that of the general public. This was important to ensure that the Ruler did not act in their own “selfish” self-interest, but rather for the good of the general public (Helvetius 1758; Wootton 2001).

While at times throughout history, economic interests were considered by some to be the most important interest, others have proposed a hierarchy of interests where individuals’ interest in intellectual pursuits, spiritual enlightenment, or relationships came secondary to “base” interests, such as survival, food and sex (Swedberg, and Agevall 2016; Lizardo and Stoltz 2017; Fineberg 1987).

We believe that the long history of disagreement about how different interests should be ranked in medicine, supports our argument that there is a need for greater nuance and flexibility when thinking about interests and COI in healthcare and biomedicine and that, in particular, assumed hierarchies of interests are problematic because they fail to account for the complex reality of clinical practice and research, and the competing demands that physicians frequently face.

### Normative assessment of interests

When first introduced, the term “interest” had negative connotations because of its association with usury and greed (Hirschman 2013a; Heilbron 2015). In the sixteenth century, however, the term came to be viewed in a positive light as it was associated with reason and rationality, and was seen to be a powerful antidote to the destructive force of the passions (Hirschman 2013a; Heilbron 2015). It was not until the late nineteenth century that the term once again came to be viewed negatively, as it was recognised that individuals’ pursuit of financial gain (which is what the term interest had then become associated with) could result in class conflict, social disruption and political upheaval (Hirschman 2013a; Heilbron 2015).

This broad normative historical trajectory was punctuated by reminders by scholars such as de La Rochefoucauld and Bentham that it is not necessary to view the pursuit of a specific interest as inherently “good” or “bad”; rather it is the behaviour that the pursuit of an interest leads to that can, at times, be problematic (Rochefoucould 1665; Bentham 1817; Swedberg 2005b). Adam Smith also reminded us that pursuit of individual interests can be good for society (Smith 1776), and Bentham described how an interest in economic gain could lead to not only greed (i.e., a negative behaviour) but also thriftiness (i.e., a positive behaviour in Bentham’s view) (Bentham 1817).

This historical trajectory has important implications for a contemporary understanding of interests in contemporary healthcare and biomedical science because it problematises the idea that interests can be viewed as negative or positive without knowing how they are enacted. Although some interests may be obviously morally questionable (such as a physician holding shares in a tobacco company), it seems problematic from a philosophical perspective to take a uniformly negative view of interests. It could be countered that even if the idea of an ‘interest’ might be morally neutral, the idea of a *conflict of* interest should be viewed negatively. However, if we reject the definition of COI based on a hierarchy of interests, it makes it difficult to argue that conflicts among the various interests are necessarily negative. For this reason, we suggest that both interests and conflicts of interest should be defined in a manner that is normatively neutral (unless perverse motives or enactments are evident) and, correspondingly, that approaches to disclosure and management should be cognisant of the complexities of professional life and the difficult trade-offs that even those with the best intentions have to make.

### Scope of interests

It is clear from a historical exploration of interests that it is not universally accepted that interests are purely economic. Even Adam Smith, who emphasised the profound societal impact of individuals’ pursuit of economic self-interest, acknowledged the role of non-financial interests, motives and drivers (Hirschman 2013a; Smith 1776). Bentham, similarly, took care to specify that multiple interests—including not only economic interest, but also an interest in food, drink, sex, power, and curiosity— could drive human behaviour (Bentham 1817). And Weber’s conceptualisation of material and ideal interests emphasised the role of interests such as those related to food, sex, survival, intellectual fulfillment, spiritual enlightenment, and relationships (Swedberg 2005a; Swedberg, and Agevall 2016; Lizardo and Stoltz 2017). It follows from this that, if it is to be grounded in the history of ideas, definitions of interests and conflicts of interests should explicitly account for non-financial interests as well as financial interests.

We believe that this analysis supports the approach to defining interests and managing conflicts of interest previously proposed by Australian philosopher and physician Paul Komesaroff and two of the authors of this paper (IK and WL) (Komesaroff et al. 2019). In a paper entitled *Conflicts of interest: new thinking, new processes*, we and Komesaroff offer the following definition:

“An interest is a commitment, goal, obligation or duty associated with a social relationship or practice” (Komesaroff et al. 2019).

A conflict of interest is then defined simply as a situation in which interests are directly in conflict with each other such that they are “likely to compel contrary and incompatible decisions, judgements or actions” (Komesaroff et al. 2019).^5^

This definition of interests and conflicts of interest has three strengths: first, it does not demand that there be a hierarchy of interests in order for a conflict of interest to exist; second, it does not assign any normative judgment to having interests or conflicts of interest; and third, it encompasses both financial and non-financial interests and COI.

In addition to offering a theoretically robust definition of interests and COI, this paper offers philosophically informed suggestions as to how interests and COIs should be identified and managed (Komesaroff et al. 2019). First, it emphasises the importance of disclosing of interests, rather than COIs, and following disclosures with robust discourse in order to determine whether a COI actually exists. In this way, it does not assume that there is a clear, pre-determined hierarchy of interests that would necessarily be recognisable to the person making the disclosure and emphasises that “the process of assessing interests should not be left to the individual himself or herself but rather must involve relevant stakeholders and be subject to the test of public scrutiny” (Komesaroff et al. 2019, p 576). Although this does not explicitly state that this is because there may need to be discussion about how different interests are ranked, the call for discourse makes such discussion possible.

The paper also provides a framework for ensuring that non-financial COIs can be disclosed without leading to a flood of meaningless declarations. To prevent lengthy disclosures of irrelevant interests, the paper outlines three criteria that should be utilised to determine whether or not an interest is material, including:

1. *Pertinence:* the interest must be relevant to the issue being considered;
2. *Substantiality:* the interest must generate substantive outcomes in the particular context;
3. *Immutability:* the interest itself, or outcomes it generates, cannot be altered through reflective deliberation or conversation (Komesaroff et al. 2019).

This approach is consistent arguments that we and others have made about non-financial COI (Wiersma et al. 2018a, b; Akl et al. 2022; Resnik 2023). For example, when it comes to religious beliefs, it has been argued that these need only be declared where they are material to a situation (Ghinea et al. 2020).

In an attempt to clearly identify interests that are likely to be material, David Resnik argues that journals should clearly specify the types of non-financial interests that researchers should disclose in the publication process in order to maintain the integrity and trustworthiness of scientific research (Resnik 2023). Resnik outlines that direct (or pertinent) research and professional interests (and relationships) ought to be disclosed, along with involvement in litigation processes, expert testimony, or acting as an unpaid scientific, technical or legal consultant (Resnik 2023). Resnik also notes that disclosure alone is insufficient and that alternative management strategies may be required if COI arise (Resnik 2023).

Eli A. Akl and colleagues classify interests according to their level (individual or institutional) and type (financial, intellectual, or personal), and outline a process for their disclosure, verification and management (Akl et al. 2022). As part of this process, they recommend conducting a risk assessment to evaluate an interest’s relevance (i.e., pertinence), nature, magnitude and recency in order to determine whether or not it creates a COI (Akl et al. 2022). If an interest does cause a COI, its management ought to be informed by what type of interest it is, and whether it represents a low, moderate or high risk of inappropriately influencing an individual’s behaviour or judgement (Akl et al. 2022).

## Practical application: physicians and the pharmaceutical and medical device industry

One of the most controversial topics in medical ethics is physicians’ relationships with the pharmaceutical industry.^6^ Physicians engage with this industry in a wide variety of ways, including having discussions with (and sometimes receiving gifts from) sales representatives, participating in industry-funded continuing medical education, receiving funding for research or participating in industry-funded research, and acting as consultants to industry. These activities have been the subject of sustained and extensive criticism from scholars in a range of disciplines including bioethics, health policy and clinical epidemiology (Grundy et al. 2018; Rodwin 2012; Purdy 2017), who have major concerns about the conflicts of interest that such activities create. When viewed in light of the history and philosophy of interests, these criticisms have three striking characteristics.

First, they focus almost exclusively on financial interests. There is now an extensive body of research showing that physicians frequently receive payments from industry (Tringale et al. 2017; Nusrat et al. 2018) and that these payments (either in the form of direct payments, travel sponsorship or meals) are associated with changes in their prescribing patterns—with industry supported physicians more likely to prescribe company branded products over generic low-cost options (Fickweiler et al. 2017; DeJong et al. 2016; Goupil et al. 2019; Brax et al. 2017). Physicians’ financial ties with industry may also bias the development of clinical guidelines (which is particularly problematic given their influence on clinical practice); (Guyatt 2010; Norris et al. 2012; Shnier et al. 2016; Neuman et al. 2011) medical education (of medical trainees and in continuing medical education) (Barnes 2017; Schofferman 2011; Golestaneh and Cowan 2017) and policymaking (Wiersma et al. 2024).

Chimonas and colleagues, in their article entitled *Mapping conflicts of interest: scoping review* found evidence of expansive financial ties between industry and health professionals across the entire healthcare ecosystem—including with research, guideline development, clinical care, and medical education providers (Chimonas et al. 2021). Physicians’ financial relationships with industry have come under increasing scrutiny in recent years with transparency efforts like the United States Physician Payments Sunshine Act^7^ and 19th version of the Medicines Australia Code of Conduct^8^ requiring doctors to disclose fees paid for educational speaking events; consulting and advisory services; sponsorship; and airfare, accommodation or registration fees (monetary amounts and precise details differ between jurisdictions) (Medicines Australia 2019; Grundy et al. 2018). These have confirmed the variety and extent of financial ties between physicians and industry. In contrast, there has been very little empirical research into the extent and impacts of non-financial interests and conflicts of interest, and few policy initiatives aimed at making these transparent (Wiersma et al. 2018a, b; Wiersma et al. 2024).

A second feature of criticisms of physicians’ interactions with industry is that they take it for granted that there is a clear hierarchy of interests, and that physicians’ financial interests should always take second place to their interests in caring for patients, conducting research, managing health budgets, and so on. (Rodwin 2012) Interests related to personal financial gain and the pharmaceutical industry, are therefore assumed to be inherently ethically problematic (Garattini and Padula 2019; Brody 2011; Stell 2010). Relatedly, critics of physicians who engage with industry also argue that the two do not share the same primary interests (Garattini and Padula 2019; Brody 2011), because the industry’s primary interest is to ensure that their profits are maximised (Garattini and Padula 2019; Brody 2011). The divergence between these two interests means that physicians’ obligations to their patients may be compromised. This means that little or no thought is given to whether there might be any circumstances in which financial interests are beneficial, or where the risks of financial conflicts of interest may be outweighed by the benefits of industry interaction.

A third, and related, feature of criticisms of physicians who interact with industry is that they are highly normative. There is an assumption that physicians engage with the pharmaceutical industry purely out of an interest in financial gain, power, and/or reputational benefits (Purdy et al. 2017; Angell 2009). Purdy and colleagues in their narrative review of debates about COI in the literature found that most articles were highly critical of physician-industry relationships, with claims made that these relationships “unconsciously and unavoidably” influence physicians, and that physicians may be “corrupted” by industry influence (Purdy 2017, p. 140).

There has been some pushback against these criticisms from those who defend doctors who engage with industry. Defenders of doctors who engage with industry claim that physicians and industry’s interests typically align—with both interested in improving patient care (Cappola and FitzGerald 2015; Stosell 2008; Stell 2010). They also claim that the term *conflict of* interest is problematic because it pejorative and presumptive of ethically problematic behaviour (Cappola and FitzGerald 2015; Stosell 2008). Rosenbaum, for example, argues in a series of controversial articles published in *The New England Journal of Medicine* that physician-industry interactions have become a moral issue—where the “sacred value of health” is perceived to have been “violated” by financially-driven industry associated physicians (Rosenbaum 2015, p 2065).

These different perspectives are evident in ongoing debates between so called “pharmascolds” who are highly critical of physicians and researchers’ relationships with industry, and so-called “pharmapologists” who defend these relationships (Garattini and Padula 2019; Brody 2011; Purdy et al. 2017; Rosenbaum 2015).

We propose that much can be gained (including potentially some common ground between ‘pharmascolds’ and ‘pharmapologists’) by reframing the debate about physicians and industry so that the full range of relevant interests (financial and non-financial) are considered; hierarchies of interests are not assumed; and strong personal judgments (positive and negative are avoided). This is not to ignore the significant body of evidence documenting the pervasive and problematic influence that industry has had on physicians (Chimonas et al. 2021), but rather to draw attention to the multiplicities of interests at play in industry-physician collaboration and the numerous ways in which these interests may coalesce (potentially benefiting patients and driving medical advancement) and converge (potentially placing patients and research participants at risk of harm).

### Scope of interests

As discussed earlier, most efforts to manage conflicts of interest in medicine, and in particular those associated with industry, have focused on financial conflicts of interest. In reality, however, it is not only financial interests that are at play here. Richard Saver points out that non-financial interests associated with physicians collaborating with industry include the possibility of enhanced reputation, career advancement, professional recognition, prestige, and access to power (Saver 2012). This assertion was supported in a qualitative interview study we conducted with Australian medical professionals, which showed that physicians relationships with industry were simultaneously viewed as a source of status, and potential source of loss of respect and stigma (Wiersma et al. 2020). While it crucial not to downplay financial interests and COIs, it seems naïve at best to simply ignore these non-financial drivers. In this regard, it is important not to make the move made by some ‘pharmapologists’, who acknowledge the existence of non-financial interests but then claim that COIs—both financial and non-financial—are so ubiquitous as to be unmanageable (Cappola and FitzGerald 2015; Stosell 2008; Stell 2010; Rosenbaum 2015).

Instead, we need to apply frameworks such as those suggested by Komesaroff et al. (2019), Ghinea et al. (2020), and Resnik (2023), that distinguish between interests (both financial and non-financial) that are pertinent, substantial and immutable and therefore material in a particular situation. If an interest is material, it should be disclosed to committee of (disinterested) peers to determine if a COI exists and how it can be managed (Komesaroff et al. 2019). The benefit of this approach is that it facilitates the management of both financial and non-financial COI, and in doing so, draws attention to the fact that non-financial interests can be a significant source of conflict, and as ethically problematic as their financial counterparts.

### Hierarchy of interests

If we question assumed hierarchies of interests, we are then able to ask when it is and is not appropriate for industry-related interests to be given priority. While it is naïve to believe that industry and physicians have perfectly aligned goals, values and interests, and goes without saying that physicians should never interact with industry in a manner that they know is likely to cause harm, there are a number of ways in which the idea of a simple hierarchy between (“secondary”) industry-related interests and other (“primary”) interests can be challenged.

For example, when a physician chooses to work with industry in a research capacity as they share an interest in discovering new medicines to treat patients with intractable illnesses (Abraham 1995). This in turn may lead to benefits for patients. Indeed, even critics of industry acknowledge that industry contribution is vital for the development of novel diagnostics, drugs and medical devices (Chimonas et al. 2021). Of course, the details of industry versus physician interests in research can diverge in significant ways—for example, industry is likely to design, interpret and disseminate studies in ways that serve their corporate interests—but this problem is not solved by creating a simple ‘industry interest/non-industry interest’ hierarchy. Rather, it is solved by recognising the subtle yet pervasive biases that can distort industry-funded research and putting in place processes to prevent or manage them. In other words, it is the specifics of behaviour in pursuit of an interest, or that arise from an interest, that determine whether or not an interest is good or bad and how it should be ranked against others.

### Normative assessment of interests

If we accept that hierarchies of interests cannot simply be assumed, then it follows that we need to be careful in the normative labels that we attach to doctors who engage with industry, as well as those that refuse to do so. The very existence of terms such as “pharmascolds” and “pharmapologists” point to a lack of normative sophistication. In this regard, we believe normative status should be assigned not based on whether or not one has a particular interest, but rather on one’s justification for pursuing that interest, how one behaves in pursuit of or as a result of it, and the context in which the person is operating. For example, Physician A, driven by a financial interest, works in an institution that promotes innovation and collaboration with industry. The institution also teaches physicians about the dangers of COI that may arise out of relationships with industry. Physician A takes pre-emptive action to ensure that their financial interests do not come into conflict with optimal care and chooses only to be involved in research that has independent oversight. Conversely, Physician B who is similarly driven by a financial interest, works in an institution that encourages any and all industry collaborations and has no governance processes in place to manage them. As a result (or perhaps simply because of their own greed), Physician B participates in sub-standard research and allows themselves to be influenced in other ways by their relationship with industry (e.g. offering patients high-cost treatments that are of little or no benefit, and in doing so, compromising their care). There seems to be a significant normative difference between these two cases, even though both physicians have the same interest.

Another factor that could be used to assign normative status is the necessity of having the interest and the likelihood that benefit will offset risk—for example, accepting money to conduct research that would not otherwise be funded seems more morally acceptable than making use of industry-funded “education” that could be provided by the profession, or accepting meals from pharmaceutical company sales reps.

With this approach in mind, strategies that use shame or stigma to deter physicians from having a COI are difficult to justify without further consideration of the nature of, reasons for, and enactment of the interest (Wiersma et al. 2020). Rather, management strategies should aim to achieve a balance between a view of COI that acknowledges that they are ubiquitous and present among all individuals, and that they can at times be ethically problematic, and may compromise patient care and physicians’ other responsibilities—including for example, ensuring the integrity of research processes.

## Conclusion

Tracing the history of the term “interests” and adopting a rich, historically informed understanding of interests in medicine has several benefits. First, it facilitates the development of a working definition of interest that acknowledges the complexity of the term, its diverse uses, and that can be used to understand the multiple influences on a physician’s behaviour. Second, it provides additional support for a nuanced approach to managing COI. This, in turn, offers a valuable lens through which to understand physicians’ behaviour, moving debates about conflicts of interest beyond pejorative accounts focused solely on physicians’ interest in financial gain, to a more nuanced understanding of the multiple interests and external factors shaping physicians’ behaviour.

## Footnotes

1 Smith uses the terms “self-interest” and “self-love” interchangeably to mean attending to one’s interest.

2 Utilitarianism is a consequentialist ethical theory that states that we ought to act in such a way that brings about the greatest good (i.e. utility), for the greatest number of people. While utility has been conceptualised in different ways, it is often viewed as happiness.

3 According to Bentham, the pursuit of an interest may be expressed in positive or negative ways. e.g. an interest in wealth may be positively expressed by an individual being frugal or negatively expressed by an individual being greedy.

4 A third position, not discussed in this paper, was that the concept of interest was not useful in sociological analysis—proposed by American sociologist Talcott Parsons.

5 We would add to this definition that interests may stem from professional or social commitments, self-interest, and relationships (Wiersma et al. 2018a, b).

6 The term “industry” is used in this paper as shorthand for the pharmaceutical and medical device industries.

7 The *US Physicians Payments Sunshine Act* was introduced in response to a Senate Inquiry into undisclosed financial payments from industry to doctors and concerns that such payments could unduly influence medical practice and research. Disclosures are made publicly available on the Open Payments website.

8 The 18th edition of the *Medicines Australia Code of Conduct* came into effect in May 2015, first addressed COI. Information is available on the Medicines Australia website and company websites.
